# Medical education-collateral damage of COVID-19?

**DOI:** 10.1136/postgradmedj-2020-138332

**Published:** 2020-07-14

**Authors:** Anni Ding

**Affiliations:** Imperial College Healthcare NHS Trust, London, UK

Dear editor,

Coronavirus Disease 2019 (COVID-19) is a potentially fatal respiratory illness, caused by a novel strain of coronavirus, SARS-CoV-2. The COVID-19 pandemic has had a profound impact globally, affecting more than 5 million people worldwide.^1^ Medical students and training doctors have been redeployed in many of the countries affected, taking on roles beyond the scope of their normal training. The move can have advantages and disadvantages for their education.

In the UK, medical school completion usually takes 5–6 years. Final examinations, in the form of written exams and Objective Structured Clinical Exams (OSCEs), are required in order to obtain a medical degree. As a result of COVID-19, some institutions have shifted forward the dates for final examinations, while many others have decided to cancel OSCEs, and opting to move written examinations to an online format. Cancellation of finals may be a disappointment to many, missing out on the opportunity to demonstrate the knowledge they have accumulated throughout medical school. For students in the middle of their medical studies, universities across the UK are completing the curriculum through remote lectures. However, much of medicine is based upon learning through patient contact, for which there is no substitute.

Due to the pressure on the National Health Service, some 5500 medical students have been brought into the workforce.^2^ Provisional registration was provided where possible to final year medical students so that they can take on Foundation Interim Year 1 roles.^2^ Students without a provisional registration have taken on roles such as porters, phlebotomists and healthcare assistants. Regardless of their stage of training, this has offered novel educational benefits beyond the scope of conventional medical school curriculum. Final year students could foreseen that their transition into a working doctor would be played out in such circumstances, this ‘baptism by fire’ has been stressful but should prepare them for their formal Foundation Year (FY) 1 role.

Many doctors, especially those working in surgical or non-patient facing specialties, have been redeployed to areas of higher demand. Redeployment brings opportunities to gain new skills, but can have its disadvantages. First, it could be argued that the new knowledge and skills gained are focused around the management of COVID-19 and therefore too niche for future use. Second, for those on specialty training programmes, the exposure to pathologies and patients representative of that specialty will be reduced. This can be particularly problematic for surgical trainees as their progression depends on achieving a required number of operations every year. The various Royal Colleges have postponed all conferences, training courses and examinations until the end of the summer at least, compounding the issues around reduced learning opportunities.

For all FY doctors, the final placement of the year has been cancelled. The Foundation Programme is designed to give junior doctors a range of experience across medical and surgical specialties that are needed to equip them with the necessary set of skills going forward, and also to spark an interest in certain specialties. Remaining in the same specialty for 8 months will reduce that breadth of exposure. As a result, some doctors may be considering taking an additional FY in order to broaden their experience. The author suggests that after the peak of the pandemic has passed, taster weeks could be used to gain insight into specialties missed out on. While this is by no means negates for the lack of rotation, it should at least compensate for the loss of experience to a small degree.

Although in these unprecedented times, patient care remains the top priority, medical education can still take place simultaneously. A significant proportion of face-to-face teaching has been replaced by virtual sessions, which can be wider-reaching as they can be accessed from any location. Interactive quizzes during virtual teaching enhance engagement^3^ and can be delivered through various platforms such as Slido and Poll Everywhere. Case-based teaching can be continued remotely as virtual Morning Reports.^4^ Open book remote examination was considered to be unchartered territory in medical education. However, they have been shown to yield similar median marks and proportion of merits and distinctions awarded as closed book examinations. Moreover, the order of questions can be randomised for individual students to mitigate against conferring.^5^ Considering the ease at which such examinations can be arranged, and the advantage of bypassing the need for a large physical venue, remote electronic examinations may well be more widely adapted in the future. Furthermore, the feasibility of replacing in-person OSCEs with remote OSCEs via Zoom has been reported.^6^ Again, this has the advantage of bypassing capacity issues and enables simultaneous assessment of more candidates, enhancing efficiency and reducing the running cost.^7^ The downside to virtual OSCEs is that physical examinations cannot be assessed. However, most other competencies can be easily assessed virtually. Small group learning via face-to-face OSCES have received positive responses; however, social distancing remains a challenge in these situations,^8^ which may preclude wider use. The power of social media has been unparalleled in these times, offering an education and discussion platform as well as mentoring opportunities.^9^

Given that the virus is unlikely to completely disappear in the foreseeable future, the medical world will inevitably have to adapt to a new way of working and educating. Moving medical education and formative assessments to a digital format has had unexpected advantages. Removing physical and logistical barriers to attending face-to-face teaching has been a welcome change to many, and in the foreseeable future, may be the easiest and safest way to ensure that regular teaching sessions can continue. As long as social distancing remains in place, remote OSCEs may be the optimal solution for practical examinations. However, for final year medical students in particular, the need for clinical exposure and physically examine patients cannot be overlooked, and they would benefit from continued clinical placements and small group face-to-face OSCEs. As William Osler once said, ‘to study the phenomenon of disease without books is to sail uncharted sea, while to study books without patients is not to go to sea at all’.

The COVID-19 pandemic represents the most significant challenge the medical world has faced in recent times. The impact on medical education may last beyond the pandemic itself. While patient care remains the top priority, the impact on students and trainees cannot be overlooked and their needs should be considered wherever possible.

## References

[1] Johns Hopkins Coronavirus Resource Center . Johns Hopkins coronavirus resource center. Available https://coronavirus.jhu.edu/ (accessed 31 Mar 2020).

[2] Dyson M . COVID-19: medical students requested to work in the NHS. *The British Medical Association is the Trade Union and Professional Body for Doctors in the UK*. Available https://www.bma.org.uk/advice-and-support/covid-19/your-contract/covid-19-medical-students-requested-to-work-in-the-nhs (accessed 13 Apr 2020).

[3] Morawo A, Sun C, Lowden M. Enhancing engagement during live virtual learning using interactive quizzes. *Med Educ*. 10.1111/medu.14253. 32438462

[4] Murdock HM, Penner JC, Le S, et al. Virtual morning report during COVID-19: a novel model for case-based teaching conferences. *Med Educ*. 10.1111/medu.14226. PMC7273056 32403168

[5] Sam AH, Reid MD, Amin A. High-stakes remote-access open-book examinations. *Med Educ*. 10.1111/medu.14247. PMC7276865 32421858

[6] Hannon P, Lappe K, Griffin C, et al. Objective structured clinical examination: from exam room to Zoom breakout room. *Med Educ*. 10.1111/medu.14241. 32418232

[7] Mooney CJ, Peyre SE, Clark NS, et al. Rapid transition to online assessment: practical steps and unanticipated advantages. *Med Educ*. 10.1111/medu.14225. 32403189

[8] Bakewell Z, Davies D, Allanby L, et al. Pandemically challenged: developing a ward-based cross-skilling programme. *Med Educ*. 10.1111/medu.14252. PMC7280587 32438448

[9] Kazerooni AR, Amini M, Tabari P, et al. Peer mentoring for medical students during COVID-19 pandemic via a social media platform. *Med Educ*. 10.1111/medu.14206. PMC7267157 32353893

